# Optimized extraction of polyphenols from rooibos tea (*Aspalathus linearis*) and their biological activities

**DOI:** 10.3389/fnut.2026.1778749

**Published:** 2026-03-06

**Authors:** Mayyas M. Othman, Bilal Azakir, Salma Khazaal, Maymouneh Rabie, Elie Salem Sokhn, Richard G. Maroun, Espérance Debs, Nicolas Louka, Nada El Darra

**Affiliations:** 1 Department of Nutrition and Dietetics, Faculty of Health Sciences, Beirut Arab University, Beirut, Lebanon; 2 Molecular and Translational Medicine Laboratory, Faculty of Medicine, Beirut Arab University, Beirut, Lebanon; 3 Department of Medical Laboratory Technology, Faculty of Health Sciences, Beirut Arab University, Beirut, Lebanon; 4 Centre d’Analyses et de Recherche, Unité de Recherche Technologies et Valorisation Agro-alimentaire, Faculté des Sciences, Université Saint-Joseph de Beyrouth, Beirut, Lebanon; 5 Department of Biology, Faculty of Arts and Sciences, University of Balamand, Tripoli, Lebanon

**Keywords:** rooibos tea, polyphenols, water bath extraction, extraction optimization, antioxidant activity, antibacterial activity, anticancer activity

## Abstract

Fermented (red) rooibos tea (*Aspalathus linearis*) has been widely consumed for its well-known health-promoting effects and represents an important dietary source of natural polyphenols. Yet, compared with unfermented (green) rooibos tea, fermented rooibos tea has received limited investigation, particularly regarding the optimization of its aqueous extraction and the systematic evaluation of its chemical composition and bioactivities. This study aimed to optimize polyphenol extraction from commercial red rooibos tea using response surface methodology (RSM), followed by assessment of the antioxidant, antibacterial, and anticancer activities of the optimized extract. In addition, the phenolic profile of the extract was characterized using ultra-high-performance liquid chromatography coupled with quadrupole time-of-flight mass spectrometry (UHPLC–QTOF–MS). The solid-to-liquid extraction using a water bath was optimized for extraction temperature and time factors. The extract was evaluated for total phenolic content (TPC) using the Folin–Ciocalteu assay, and for antioxidant activity using the DPPH (2,2-diphenyl-picrylhydrazyl) assay. RSM analysis indicated that temperature was the dominant factor influencing both TPC and DPPH activity, with time showing a secondary temperature-dependent effect. At the optimized conditions (90 °C, 10 min), the optimum rooibos tea extract (RTE) showed a TPC of 49.94 mg GAE/g DM and an antioxidant activity of 101.90 mg TE/g DM. UHPLC–QTOF–MS analysis identified 43 metabolites in the RTE, mainly isoorientin, rutin, aesculetin, and aspalathin among others. The RTE produced inhibition zones of 12 mm against *Staphylococcus aureus* and 10 mm against *Pseudomonas aeruginosa* in the disc diffusion assay, while no inhibition zones were observed for *Bacillus cereus*, *Listeria monocytogenes*, *Escherichia coli*, or *Salmonella Typhimurium*. Broth microdilution assays yielded Minimum Inhibitory Concentration/Minimum Bactericidal Concentration (MIC/MBC) values of 25/25 mg/mL for *S. aureus*, 25/50 mg/mL for *L. monocytogenes*, 50/50 mg/mL for *P. aeruginosa*, and 50/100 mg/mL for *E. coli*, whereas *B. cereus* and *S. typhimurium* showed resistance to RTE under the tested conditions. For the anticancer activity, evaluated using MTT (3-(4,5-dimethylthiazol-2-yl)-2,5-diphenyltetrazolium bromide) assay, the extract did not achieve 50% inhibition across the concentrations tested (50–700 μg/mL) against H460, HT-29, and Caco-2 cancer cell lines. This study is the first to integrate RSM-based optimization of aqueous red rooibos extraction with chromatographic profiling and *in vitro* biological assays. Future studies may compare red and green rooibos tea, and explore product variability, *in vivo* relevance, and alternative processing strategies.

## 1 Introduction

Rooibos tea (made from the *Aspalathus linearis* herb) is an increasingly popular South African herbal infusion with a long history of use for both its ethnic significance, and its perceived health benefits largely attributed to its phytochemical composition (1–4). Endemic to the Cape Floristic Region of South Africa (5), rooibos tea is naturally caffeine free and low in tannins compared with *Camellia sinensis* teas. This fact has contributed to its growing appeal among health-conscious consumers and aligns with the global interest in natural products with functional properties (3, 6, 7).

Rooibos tea is produced in two forms, red (fermented) and green (unfermented), with the difference lying mainly in processing. Red rooibos undergoes cutting, bruising, and controlled oxidation (8), a step that alters two key C-glycosylated flavonoids of rooibos, aspalathin and nothofagin. Most of the aspalathin, found exclusively in the rooibos plant (8), is enzymatically converted during fermentation into flavone derivatives such as isoorientin, and can further polymerize into high-molecular-weight brown compounds responsible for the characteristic color of red rooibos (9–12). Nothofagin, although more stable, also undergoes enzymatic and chemical degradation, giving rise to additional secondary phenolic products during fermentation (8). In contrast, green rooibos does not undergo an oxidation step and consequently retains higher levels of its native polyphenols (8).

Although green rooibos generally (but not always) shows stronger antioxidant activity due to its higher retention of the native flavonoids (13–15), fermentation does not diminish the functional value of the red variety (16). The oxidative changes that occur during processing generate a mixture of phenolic compounds that collectively contribute to the antioxidant potential of red rooibos, allowing it to retain notable activity and a documented variety of biological effects (17–25). Moreover, red rooibos remains the most widely consumed and commercially established form, making it especially more relevant for research on its health-promoting properties (8, 26).

The antioxidant potential of red rooibos has been demonstrated in both animal and human studies. In rat models, red rooibos intake consistently reduced oxidative damage, and improved overall antioxidant status under normal conditions, during psychological stress, and in chemically induced liver injury (27–29). In humans, consumption of red rooibos led to a rapid increase in circulating antioxidant capacity following ingestion (16). Beneficial effects were also reported in individuals chronically exposed to lead, where regular rooibos intake reduced oxidative damage and improved antioxidant balance despite ongoing toxic exposure (30).

Beyond its antioxidant function, red rooibos demonstrates additional bioactivities. Although the antibacterial potential of red rooibos has been less extensively studied than its other properties, several reports suggest that it may exert inhibitory effects against a range of bacterial species. For example, rooibos extracts have been reported to demonstrate activity against *Micrococcus luteus* and *Bacillus cereus* on solid media (31). Comparative work further suggests that red rooibos often displays stronger inhibitory effects than the green form, with activity increasing in a concentration-dependent manner (24). Importantly, artificial infusions prepared only from known rooibos polyphenols did not reproduce the antibacterial effects of natural extracts, indicating that the activity cannot be attributed exclusively to identified flavonoids and may instead involve synergistic or non-phenolic constituents (24).

Furthermore, although evidence is limited, some studies show that red rooibos can exert anticancer effects. In a mouse skin carcinogenesis model, topical application of red rooibos extracts significantly suppressed tumor formation, achieving a 75% reduction in tumor yield, an effect attributed to the flavonoid composition and non-phenolic constituents present after fermentation (25). Similar protective trends were observed in a rat model of liver cancer promotion, where red rooibos significantly reduced oxidative damage (32). *In vitro*, aqueous red rooibos extracts demonstrated dose-dependent cytotoxicity against colon (HCT-116), prostate (PC-3), and liver (HepG2) cancer cell lines (33).

Despite substantial interest, systematic optimization of polyphenol extraction from red rooibos remains limited. Extraction efficiency can vary widely depending on the extraction method, solvent composition, temperature, time, solid–liquid ratio, and other process parameters, all known to affect phenolic recovery in plant matrices (34–37). Nevertheless, studies on red rooibos continue to rely on non-optimized or inconsistent conditions, making comparisons difficult (24, 31, 33). In this context, statistical optimization techniques, such as response surface methodology (RSM), can be used to systematically evaluate multiple extraction factors and identify optimal conditions (38, 39). Moreover, advanced extraction techniques such as microwave-assisted extraction, ultrasound-assisted extraction, and others may provide higher extraction yields of polyphenolic compounds from plant matrices compared with conventional techniques (40–42). However, conventional water bath extraction remains the most widely used for teas due to its simplicity, affordability, and relevance to how the tea is consumed (43, 44). Importantly, short-time extraction at high temperatures using this traditional method can preserve polyphenol structures essential for biological activity (45). Furthermore, while organic solvents can extract polyphenols efficiently, safety concerns, toxicity, and the need for additional purification limit their suitability for food-related applications (46, 47). On the other hand, water is highly suitable for rooibos tea since its major phenolics are hydrophilic, making water usage as a solvent both a practical and representative extraction medium (4, 48–50).

Beyond extraction, gaps also persist in the comprehensive evaluation of red rooibos bioactivity. Much of the recent focus has centered on green rooibos, while studies on the red form rarely integrate optimized extraction with subsequent assessment of antioxidant, antibacterial, and anticancer properties. Moreover, detailed chromatographic profiling is rarely paired with biological testing, limiting the ability to make important connections regarding the relationships between extraction conditions, phenolic composition, and bioactivity. To address these gaps, the present study aims to optimize the water bath extraction of polyphenols from red rooibos tea using RSM and to evaluate the antioxidant, antibacterial, and anticancer activities of the optimized extract. Comprehensive chromatographic profiling using UHPLC–QTOF–MS is further employed to characterize the phenolic composition and relate it to the observed bioactivities. By integrating extraction optimization under conditions relevant to typical tea preparation with detailed chemical profiling and *in vitro* bioactivity assessment, this work provides a more holistic evaluation of red rooibos tea than currently available. To the best of our knowledge, this is the first study to systematically assess the antibacterial activity of aqueous red rooibos extract using both disc diffusion and MIC/MBC assays across a broad bacterial panel, as well as the first to investigate its effects on H460, HT-29, and Caco-2 cell lines *in vitro*.

## 2 Materials and methods

### 2.1 Raw materials

Commercial red rooibos (*Aspalathus linearis*) tea produced by Carmién Tea (South Africa) was purchased online. The product consisted of 2.5 g teabags containing finely cut, fermented rooibos with a reddish-brown color representative of the commercial rooibos tea available to consumers. To our knowledge, specific fermentation parameters were not disclosed by the manufacturer. The purpose of using a commercial product is to better reflect the quality and characteristics of rooibos teas currently circulating on the market. All teabags were kept in their original sealed packaging and stored in a cool, dry environment until extraction.

### 2.2 Chemicals, reagents, and media

All chemicals and reagents used in this study were of analytical grade. For the total phenolic content (TPC) and DPPH (2,2-diphenyl-picrylhydrazyl) antioxidant activity assays, Folin–Ciocalteu reagent was purchased from Scharlau (Barcelona, Spain); sodium carbonate (Na_2_CO_3_) from Merck (Darmstadt, Germany); gallic acid (3,4,5-trihydroxybenzoic acid) standard from Ambeed (Arlington Heights, IL, United States); and trolox (6-hydroxy-2,5,7,8-tetramethylchromane-2-carboxylic acid) from Biosynth Carbosynth (Compton, United Kingdom). DPPH and methanol were sourced from Sigma-Aldrich (Steinheim, Germany).

For antibacterial assays, several microbiological media were used. Plate Count Agar, MacConkey Agar, Mueller–Hinton Broth, Mannitol Salt Agar Base, and Blood Agar Base Infusion were obtained from HiMedia (Mumbai, India). Listeria Agar Base (Oxford formulation) was purchased from Pronadisa Conda (Madrid, Spain). Cetrimide Agar, Nutrient Broth, and Mueller–Hinton II Agar were supplied by Liofilchem (Roseto degli Abruzzi, Italy).

For the anticancer assay, RPMI-1640, Dulbecco’s Modified Eagle Medium (DMEM), trypan blue stain, and fetal bovine serum (supplement) were sourced from Sigma-Aldrich (Steinheim, Germany). Penicillin–streptomycin (supplement) was obtained from GeneDirex (Taipei, Taiwan).

### 2.3 Dry matter determination

Dry matter (DM) content was determined by placing the tea in a hot air oven to dry at 105 °C for 24 h, then reweighed (51–53). Analyses were performed in triplicate. The resulting DM content was subsequently determined and expressed as a percentage, which was found to be 99.625% *w/w*.

### 2.4 Extraction methods

#### 2.4.1 Extraction procedure

Polyphenols were extracted from rooibos tea using a solid-to-liquid water bath extraction method. Briefly, 1 g of red rooibos tea (taken from the commercial teabag) was added to preheated distilled water at the required solid-to-liquid ratio and incubated at the designated temperature for a specified extraction time in a water bath (DKZ-1 series, Shanghai Lilang Scientific Instrument Co., Ltd., Shanghai, China). Following extraction, the mixtures were filtered through glass wool, then centrifuged at 4500 rpm for 10 min (54) using a Centurion Scientific C2 Series centrifuge (Lancing, United Kingdom). The resulting supernatants were collected and stored at −20 °C until further analysis.

#### 2.4.2 Determination of optimal solid-to-liquid ratio

A range of solid-to-liquid ratios, from 1/10 to 1/100 g/mL, was tested for the extraction process of rooibos tea at a set temperature and time. Once the solid-to-liquid ratio yielding the most efficient TPC was determined, RSM was applied to optimize the time and temperature extraction conditions.

### 2.5 Experimental design

RSM was applied to evaluate both the individual and interactive effects of key extraction parameters. Extraction temperature (T) and time (t) were selected as the independent variables influencing TPC and antioxidant activity measured by the DPPH assay.

A central composite experimental design was implemented, consisting of 12 experimental runs including four repetitions at the center point. Extraction temperatures ranged from 60 to 90 °C, while extraction times varied between 10 and 40 min, with coded levels assigned as −1 and +1 for the minimum and maximum values, respectively.

The experimental data were modeled using a second-order polynomial equation to describe the relationship between the response and the independent variables:


$$Y=\alpha_{0}+\alpha_{1}\times T+\alpha_{2}\times t+\alpha_{3}\times T^{2}+\alpha_{4}\times T\times t+\alpha_{5}\times t^{2}$$

*Y* denotes the estimated response variable, corresponding to either TPC or DPPH scavenging activity; α_0_ reflects the mean response at the center point; α_1_ and α_2_ represent the individual effects of extraction temperature and duration, respectively, α_3_ and α_5_ represent nonlinear (quadratic) influences of these factors; α_4_ represents the interactive term between temperature and time.

### 2.6 Determination of TPC

TPC of the rooibos tea extract (RTE) was quantified using the Folin–Ciocalteu colorimetric method (53, 55). 500 μL Folin–Ciocalteu reagent (diluted 1/10 *v/v*) and 400 μL Na_2_CO_3_ (7.5% *w/v*) were added to 100 μL of the RTE. The mixture was vortexed then incubated at 60 °C for 10 min, then cooled at 4 °C for 10 min, prior to measurement at 750 nm using a UV–Vis spectrophotometer (Optima, Tokyo, Japan).

Gallic acid (0.00039–0.5 mg/mL) was used to establish a calibration curve (R^2^ ≥ 0.99). Results were expressed as mg gallic acid equivalents per gram dry matter (mg GAE/g DM).

### 2.7 Determination of antioxidant activity

Antioxidant activity of the RTE was assessed by measuring the ability of polyphenols present in the extract to neutralize the stable DPPH radical (56, 57). 1.45 mL of freshly prepared DPPH solution (0.06 mM) was added to 50 μL of the RTE. The reaction mixture was allowed to stand for 30 min at room temperature in the dark. The negative control consisted of 50 μL of methanol mixed with 1.45 mL of DPPH solution. Absorbance was then measured at 515 nm, with methanol as the blank, using a UV–Vis spectrophotometer (Optima, Tokyo, Japan). Antioxidant activity was determined using the formula:


$$\text{Inhibition of DPPH}\;(\%)=\frac{\mathrm{Abs}\;(\text{negative control})-\mathrm{Abs}\;(\text{sample})}{\mathrm{Abs}\;(\text{negative control})}\times 100$$

Antioxidant activity of RTEs was ultimately reported as milligrams of Trolox equivalents per gram of dry matter (mg TE/g DM).

### 2.8 Freeze-drying of RTE

The RTEs were first frozen at −80 °C. Then, lyophilization was carried out using a Telstar freeze dryer (Barcelona, Spain) at the Faculty of Sciences in Beirut Arab University, operating at −50 °C under a vacuum of 0.1 mbar. The resulting powders were preserved at −20 °C until further use. The average yield across three independent lyophilization runs was 8.81%.

### 2.9 UHPLC-QTOF-MS analysis of RTE for phytochemical characterization

The phytochemical profile of the RTE was characterized using ultra-high-performance liquid chromatography coupled with quadrupole time-of-flight mass spectrometry (UHPLC–QTOF–MS), as previously described by Helmi et al. (58). Analyses were performed using a Bruker Daltonik Elute UHPLC system (Bremen, Germany) coupled to a Bruker Impact II ESI-QTOF mass spectrometer equipped with an Apollo II electrospray ionization source. The system was operated in both positive and negative electrospray ionization (ESI) modes to allow comprehensive detection of compounds with different ionization behaviors. Chromatographic separation was achieved on a Bruker Solo 2.0 C18 column (100 mm × 2.1 mm × 2.0 μm), maintained at 40 °C. The mobile phase consisted of water containing 0.01% formic acid (A) and acetonitrile (B), delivered at a flow rate of 0.51 mL/min over a total run time of 35 min for each ionization mode. The injection volume was 3 μL. Prior to analysis, 10 mg of powdered RTE was dissolved in 100 μL dimethyl sulfoxide (DMSO) and diluted with 900 μL of methanol. A 250 μL aliquot of this solution was further diluted with 1 mL methanol, centrifuged at 4000 rpm for 2 min, and 1 mL of the resulting supernatant was transferred to autosampler vials. Mass spectrometric conditions included a capillary voltage of 2,500 V, nebulizer pressure of 2.0 bar, dry nitrogen gas flow of 8 L/min, and a drying temperature of 200 °C. Data were acquired at a mass resolution of 50,000 FSR with a TOF repetition rate of up to 20 kHz, and mass accuracy was maintained below 1 ppm. Compound identification was carried out by comparing accurate mass measurements and retention times with those obtained from authentic reference standards prepared in DMSO and acetonitrile. The standards are listed in Supplementary Table S1. All solvents and reagents used were of analytical grade to ensure accuracy and minimize background interference.

### 2.10 Assessment of antibacterial activity

#### 2.10.1 Bacterial strains

Six bacterial strains were tested, including three Gram-positive strains (*Staphylococcus aureus* ATCC 25923, *Bacillus cereus* clinical isolate, *Listeria monocytogenes* ATCC 19115) and three Gram-negative strains (*Escherichia coli* ATCC 25922, *Salmonella Typhimurium* ATCC 14028, *Pseudomonas aeruginosa* clinical isolate). *E. coli*, *B. cereus*, and *S. aureus* were supplied by the Medical Lab Department at the Faculty of Health Sciences at Beirut Arab University, while *S. typhimurium*, *P. aeruginosa*, and *L. monocytogenes* were supplied by Lebanese Agricultural Research Institute, Fanar, Lebanon. All bacterial strains were preserved in glycerol broth and stored at −20 °C until use.

#### 2.10.2 Standardization of bacterial inoculum

Uniform and well-defined bacterial colonies were aseptically collected from respective agar plates and suspended in sterile Mueller-Hinton Broth, followed by thorough mixing to obtain a homogenous suspension, and standardized to the 0.5 MacFarland standard (1.5 × 10^8^ CFU/mL) by measuring the optical density (0.08–0.13) at 600 nm using a UV–Vis spectrophotometer (Optima, Tokyo, Japan) (59).

#### 2.10.3 Disc diffusion assay

The antibacterial potential of the RTE was first assessed using the disc diffusion technique (53, 60). The standardized bacterial suspensions were subsequently diluted 100-fold to obtain an approximate final concentration of 1.5 × 10^6^ CFU/mL. Mueller–Hinton agar plates were evenly inoculated by spreading the diluted suspension across the surface with a sterile cotton swab, rotating the plate by 60° between streaks and finishing with swabbing along the plate perimeter.

The powder RTE was reconstituted in distilled water to obtain a solution of 100 mg/mL, which was subsequently sterilized by passage through a 0.22 μm syringe filter. Sterile paper discs (6 mm diameter) were then positioned on the agar surface and loaded with 20 μL of RTE. Discs containing 20 μL of distilled water served as negative controls, whereas discs with 20 μL antibiotic solutions (gentamicin and imipenem) were used as positive controls.

Following incubation at 37 °C for 24 h, antibacterial activity was quantified by measuring the diameters of the clear inhibition zones surrounding each disc, expressed in millimeters.

#### 2.10.4 Determination of minimum inhibitory concentration

The minimum inhibitory concentration (MIC) of the RTE against the tested bacterial strains was established using the broth microdilution assay (61, 62). After preparing the primary stock solution of 100 mg/mL of the RTE, which was filtered through a 0.22 μm syringe filter for sterilization, a series of two-fold dilutions was done to yield final extract concentrations (50, 25, 12.5, 6.25, 3.125, 1.5625, 0.78125, and 0.390625 mg/mL). For the assay, sterile 96-well microtiter plates were used, with 100 μL of standardized bacterial suspension mixed with 100 μL of each extract dilution per well, resulting in a final bacterial concentration of 7.5 × 10^5^ CFU/mL. Control wells containing only bacterial suspension with broth medium were included to check normal microbial growth, while wells containing extract and broth medium without bacteria were used to correct for any interference caused by the natural coloration of the extract. Additional wells containing broth alone served as sterility controls. The microplates were sealed and incubated at 37 °C for 24 h. The MIC was defined as the lowest extract concentration at which visible bacterial growth was completely inhibited when compared with the appropriate control wells, in accordance with CLSI guidelines (63).

#### 2.10.5 Determination of minimum bactericidal concentration

The minimum bactericidal concentration (MBC) of the RTE was subsequently determined by subculturing 100 μL from wells corresponding to the MIC and higher concentrations onto Plate Count Agar. The inoculated plates were incubated at 37 °C for 24 h, alongside broth and agar controls to confirm assay sterility. The MBC was defined as the lowest extract concentration producing a ≥ 99.9% reduction in viable bacterial counts compared with the initial inoculum (63).

### 2.11 Determination of anticancer activity

#### 2.11.1 Cell culture and treatment

The anticancer activity of the RTE was assessed against the Caco-2 (colon carcinoma), HT-29 (colorectal adenocarcinoma), and H460 (non-small cell lung carcinoma) cancer cell lines (gift from Professor Salem Chouhaib, Gulf Medical University). Caco-2 cells were cultured in DMEM (Dulbecco’s Modified Eagle Medium), whereas H460 and HT-29 cells were cultured in RPMI 1640. Both media were supplemented with 10% FBS and 1% penicillin–streptomycin. Cells were incubated at 37 °C, 5% CO_2_ and 20% O_2_.

#### 2.11.2 MTT assay

The anticancer effects of the RTE were examined *in vitro* using the 3-(4,5-dimethylthiazol-2-yl)-2,5-diphenyltetrazolium bromide (MTT) colorimetric assay, which evaluates cellular metabolic activity as an indicator of viability (64, 65). The cancer cells were seeded in 96-well plates at densities optimized for each model (Caco-2: 80,000 cells/mL; HT-29: 120,000 cells/mL; H460: 60,000 cells/mL), with 100 μL dispensed per well. Plates were incubated at 37 °C and 5% CO_2_ for 24 h to allow cell attachment prior to treatment. After the incubation period, the culture medium was removed and replaced with 100 μL of RTE working solutions at concentrations of 50, 100, 200, 400, 600, and 700 μg/mL, prepared from a fresh 1 mg/mL stock solution diluted with sterile distilled water. Control wells contained cells maintained in their respective complete culture media without extract. Following 72 h treatment, the medium was aspirated and replaced with 100 μL of 10% MTT working solution, which was prepared by dissolving 0.1 g of MTT powder in 20 mL of PBS, and subsequently mixing 2.5 mL of this solution with 22.5 mL of the corresponding complete media. Plates were incubated for 2 h to allow the formation of insoluble formazan crystals by metabolically active cells. The MTT solution was then removed, and 100 μL of DMSO was added to each well to solubilize the crystals. Plates were gently agitated for 10 min using an orbital shaker, and absorbance was measured at 570 nm using a microplate reader (Thermo Fisher Scientific, Boston, MA, USA). Background absorbance from blank wells was subtracted from all measurements. Cell viability was expressed as a percentage using the following equation:


$$\text{Cell viability}\;(\%)=\frac{\text{Absorbance of treated cells}}{\text{Absorbance of control cells}}\times 100$$

### 2.12 Statistical analysis

All assays were performed independently in triplicates (*n* = 3), and the data are reported as mean values accompanied by their corresponding standard deviations (SD). Data organization, calculation of means and errors, and graphical representations (e.g., bar charts) were performed using Microsoft Excel (Microsoft Corp., Redmond, United States). The half-maximal inhibitory concentration (IC_50_) values obtained from anticancer experiments were calculated by nonlinear regression curve fitting using MicroCal Origin software (OriginLab Corp., Northampton, USA; version 6, 1991–1999). Statistical significance was considered at *p* < 0.05.

## 3 Results and discussion

### 3.1 Determination of optimum solid-to-liquid ratio for extraction

To establish an efficient solid-to-liquid ratio for the extraction of polyphenols from red rooibos tea, several ratios ranging from 1:10 to 1:100 g/mL, were tested and compared based on their TPC, with the results presented in Table 1. Optimizing this parameter is important, because the solvent volume directly influences the concentration gradient between the plant material and the extracting medium, thereby affecting mass transfer, and phenolic diffusion (58).

**Table 1:** Average TPC at various solid-to-liquid ratios of RTE tested.

Solid-to-liquid ratio (g/mL)	TPC (mg GAE/g DM)
1:10	11.12 ± 0.14^a^
1:20	19.89 ± 0.77^b^
1:30	27.96 ± 0.48^c^
1:40	28.85 ± 0.49^c^
1:50	35.92 ± 0.71^d^
1:60	48.87 ± 0.57^e^
1:100	51.10 ± 0.37^e^

Values are expressed as mean ± standard deviation (*n* = 3). Values within the same column followed by different superscript letters are significantly different (*p* < 0.05).

The results demonstrated a clear increase in TPC of RTE with increasing solvent volume. TPC increased notably from 11.12 mg GAE/g DM at a ratio of 1:10 to 48.87 mg GAE/g DM at 1:60. Beyond this point, only a relatively minor increase was observed, with TPC reaching 51.10 mg GAE/g DM at 1:100, suggesting that further solvent addition provided a limited benefit. This indicates that the majority of the extractable phenolic compounds had already been solubilized at the 1:60 ratio, and that the system reached equilibrium after.

Similar extraction behavior has been widely reported in literature. Increasing solvent volume initially enhances the phenolic recovery by facilitating diffusion and improving mass transfer. However, beyond a certain point, the system approaches extraction equilibrium, and further increases in solvent do not result in additional phenolic release (52, 66, 67). Consequently, the solid-to-liquid ratio of 1:60 was selected for the subsequent experiments.

### 3.2 Effect of time and temperature on TPC and DPPH of RTE

RSM was employed to determine the optimal operating conditions for the extraction with the aim of maximizing TPC and DPPH antioxidant activity. For all 12 runs, the solid-to-liquid ratio was fixed at 1:60 g/mL, while the extraction temperature and time were systematically varied to construct the predictive model.

The experimental design and corresponding responses are shown in Table 2. The extraction temperature and time were varied over a range of 53.8–96.2 °C and 3.8–46.2 min, respectively, according to the central composite design. Across the tested conditions, TPC values ranged from 39.29 to 62.56 mg GAE/g DM, while DPPH varied between 85.48 and 106.55 mg TE/g DM. Repeated center-point experiments (75 °C, 25 min) showed good reproducibility, as evidenced by low variability in both TPC and DPPH. TPC values showed a mean of 50.02 ± 0.99 mg GAE/g DM, while DPPH averaged 99.79 ± 1.68 mg TE/g DM, indicating a high level of experimental consistency at the center point.

**Table 2:** Central composite design for the independent variables (temperature and time) and the corresponding responses of RTE for TPC (mg GAE/g DM) and DPPH (mg TE/g DM).

Design points		Runs	Extraction parameters		Responses	
	Temperature (°C)		Time (min)	TPC (mg GAE/g DM)	DPPH (mg TE/g DM)
Factorial Points	1	60 (−1)	10 (−1)	39.29	86.17
	2	90 (+1)	10 (−1)	47.63	106.55
	3	60 (−1)	40 (+1)	44.98	99.70
	4	90 (+1)	40 (+1)	59.44	99.53
Star points	5	53.8 (−α)	25 (0)	43.33	85.48
	6	96.2 (+α)	25 (0)	62.56	99.02
	7	75 (0)	3.8 (−α)	49.19	103.98
	8	75 (0)	46.2 (+α)	53.53	103.81
Center points	9	75 (0)	25 (0)	48.71	101.76
	10	75 (0)	25 (0)	49.80	100.39
	11	75 (0)	25 (0)	50.84	99.19
	12	75 (0)	25 (0)	50.71	97.82

**Figure 1:**
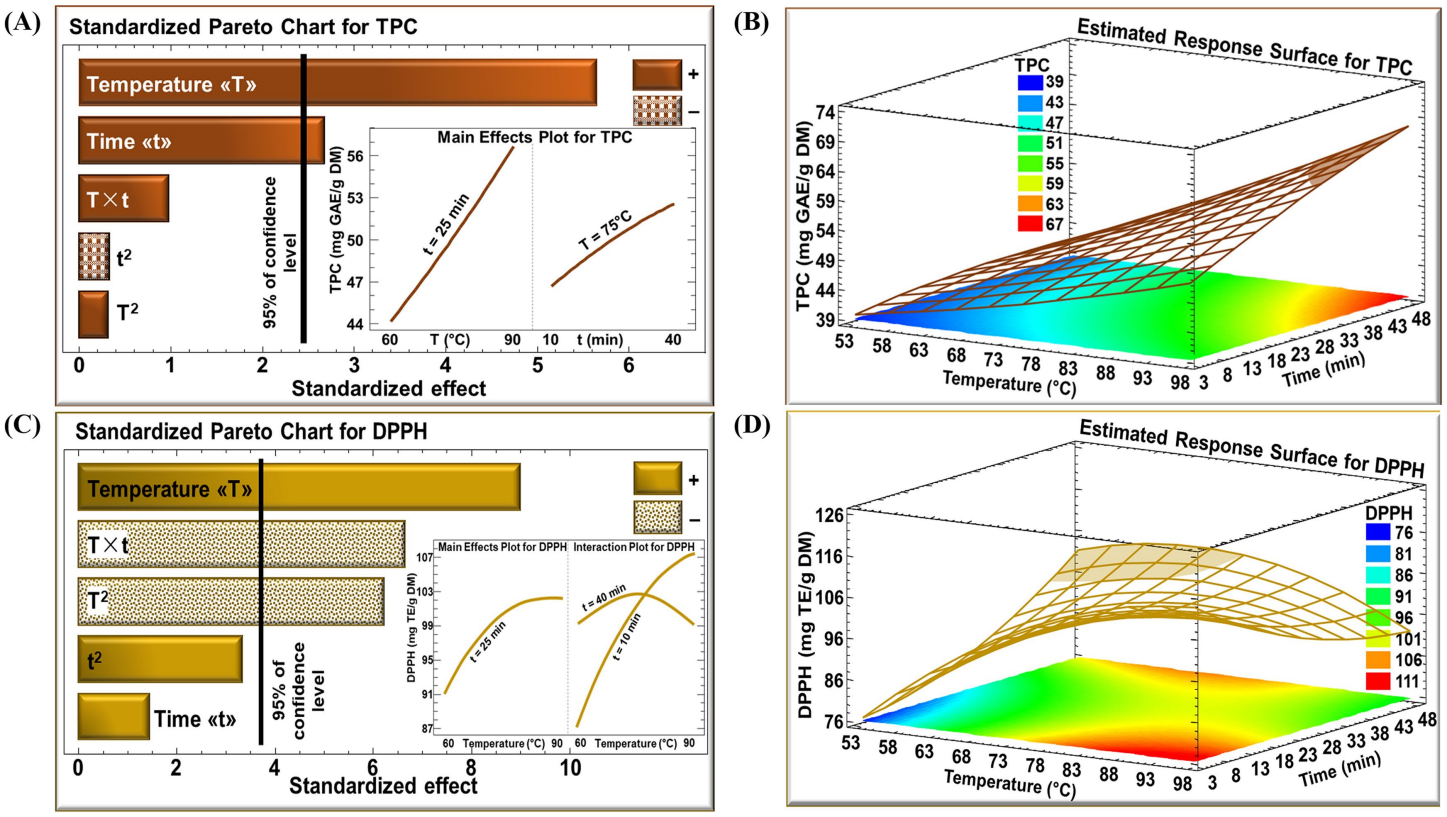
Standardized Pareto chart with insert for the effect of temperature and time on RTE for **(A)** TPC and **(C)** DPPH, and estimated response surface for **(B)** TPC and **(D)** DPPH. (+) Denotes a positive effect and (−) denotes a negative effect.

The effects of time and temperature variables on TPC and DPPH activity were evaluated using Pareto analysis and 3D response surface plots, illustrated in Figure 1. In the Pareto charts, effects are considered statistically significant (*p* < 0.05) when the corresponding horizontal bars extend past the reference threshold line.

The standardized Pareto chart for TPC (Figure 1A) demonstrates that extraction temperature had the strongest positive linear influence on phenolic recovery. When extraction time was held constant at 25 min, increasing the temperature from 60 °C to 90 °C resulted in a notable increase in TPC, from 44 to over 56 mg GAE/g DM (insert of Figure 1A). Extraction time also showed a positive effect, though of lower extent, with TPC increasing from 47 to 52 mg GAE/g DM as time increased from 10 to 40 min at a constant temperature of 75 °C (insert of Figure 1A). These trends are further confirmed by the response surface plot (Figure 1B), which shows a high-response region representing optimal conditions for maximizing TPC which reached around 67 mg GAE/g DM (indicated by the brown zone).

The Pareto chart for DPPH (Figure 1C) revealed that temperature had a significant positive linear effect, with DPPH values increasing from 91 mg TE/g DM at 60 °C to 103 mg TE/g DM at 80 °C (insert of Figure 1C). Beyond this point, a significant negative quadratic effect of temperature was observed, which reduced the antioxidant activity. This suggests the existence of an optimal temperature range for maximizing antioxidant activity beyond which further temperature increases become detrimental. The interaction term between temperature and time (T × t) was also significantly negative, indicating that the two variables jointly influenced antioxidant activity rather than acting independently. As illustrated in the insert of Figure 1C, at a short extraction time (10 min), increasing the temperature from 60 to 90 °C resulted in a pronounced increase in DPPH values (from approximately 87 to 107 mg TE/g DM). In contrast, at a longer extraction time (40 min), the influence of temperature was attenuated, with DPPH activity increasing initially but subsequently decreasing, ultimately returning to values close to the initial level (around 100 mg TE/g DM). The corresponding response surface (Figure 1D) identifies two distinct regions (indicated by yellow zones) of maximal DPPH activity (111 mg TE/g DM), one at high temperature with short extraction times (80–98 °C and 3–10 min), and another at lower temperature combined with extended extraction time (around 70 °C at an average and 48 min).

Temperature was the dominant factor influencing both TPC and DPPH of the extract. Increasing the extraction temperature enhanced the release of phenolic compounds from the rooibos tea. This effect is attributed to improved mass transfer at elevated temperatures, driven by reduced solvent viscosity and surface tension, as well as heat-induced changes in diffusivity and matrix permeability, which facilitates solute diffusion (68, 69). However, beyond a critical thermal threshold, phenolic degradation and oxidative polymerization may occur, leading to reduced antioxidant yield and capacity (45, 70). Similar heat-dependent trends have been reported for red rooibos and other herbal extracts, confirming temperature as a key determinant of polyphenol recovery (71).

Extraction time influenced TPC and DPPH responses differently and to a lesser extent than temperature. For TPC, extraction time showed a positive linear contribution, indicating that extended contact between solvent and plant matrix enhanced phenolic solubilization. This suggests that longer extraction primarily facilitates diffusion-controlled release of phenolics rather than limiting recovery. Similar observations have been reported where time plays a secondary but supportive role in maximizing phenolic yield under aqueous extraction conditions (52, 72).

In contrast, the effect of extraction time on DPPH was strongly dependent on temperature, as reflected by the significant interaction between these variables. While longer extraction times were beneficial at lower temperatures, prolonged exposure at elevated temperatures was associated with reduced DPPH, likely reflecting increased susceptibility of antioxidant constituents to thermal or oxidative transformations (44, 73). Previous studies similarly indicate that moderate extraction times, tailored to the applied temperature, provide an optimal compromise between antioxidant preservation and release (51, 74, 75).

RSM enabled the development of second-order polynomial models describing the relationships between extraction parameters and response variables. The regression equations for TPC and DPPH activity, including both full, and simple (reduced) models after elimination of nonsignificant terms (76), are shown in Table 3. A good fit between the experimental and predicted values was observed, with the models showing satisfactory R^2^ levels that indicate reasonable agreement (77).

**Table 3:** Second-order regression equations for TPC and DPPH activity for RTE generated by RSM.

Model	Equation	R^2^
Full	$\mathrm{TPC}=35.1-0.01T-0.22t+0.002T^{2}+0.007\mathrm{Tt}-0.002t^{2}$	0.910
Simple	TPC=13.8+0.42T−0.19t	
Full	$\text{DPPH}=-58.+3.4T+1.3t-0.017T^{2}-0.023\mathrm{Tt}+0.009t^{2}$	0.969
Simple	$\text{DPPH}=-70.93+3.7T+1.7t-0.019T^{2}-0.023\mathrm{Tt}$	

(1) and (3) are the full model equations while (2) and (4) are the simplified equations having only the significant parameters.

**Figure 2:**
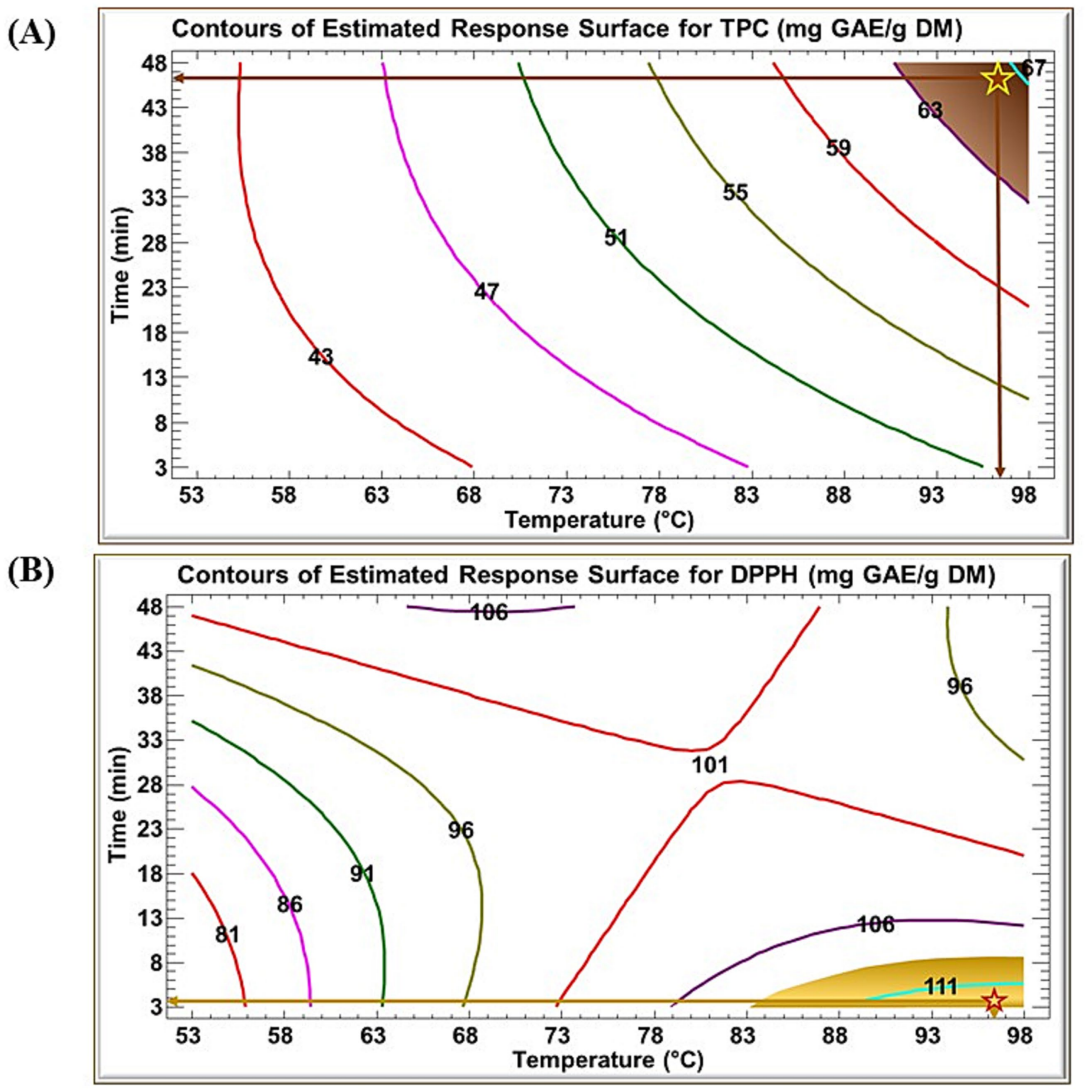
Contours of estimated response surface for **(A)** TPC and **(B)** DPPH of RTE as a function of time and temperature.

Figure 2 presents the contour plots of the estimated response surfaces for TPC (Figure 2A) and DPPH (Figure 2B). Each contour line represents a constant predicted response value, while progression from outer to inner contours corresponds to increasing response magnitude (i.e., higher TPC or higher DPPH). Accordingly, regions enclosed by contours with larger numerical values indicate more favorable temperature–time combinations for maximizing the respective response.

Figure 2A shows that the predicted optimum conditions for maximizing TPC were 96.21 °C and 46 min, corresponding to a theoretical TPC of 66.05 mg GAE/g DM (Figure 2A). In contrast, the highest predicted DPPH activity (112.4 mg TE/g DM) was obtained at 96.2 °C and 3.7 min (Figure 2B), meaning that the absolute optima for the two responses did not coincide.

**Figure 3:**
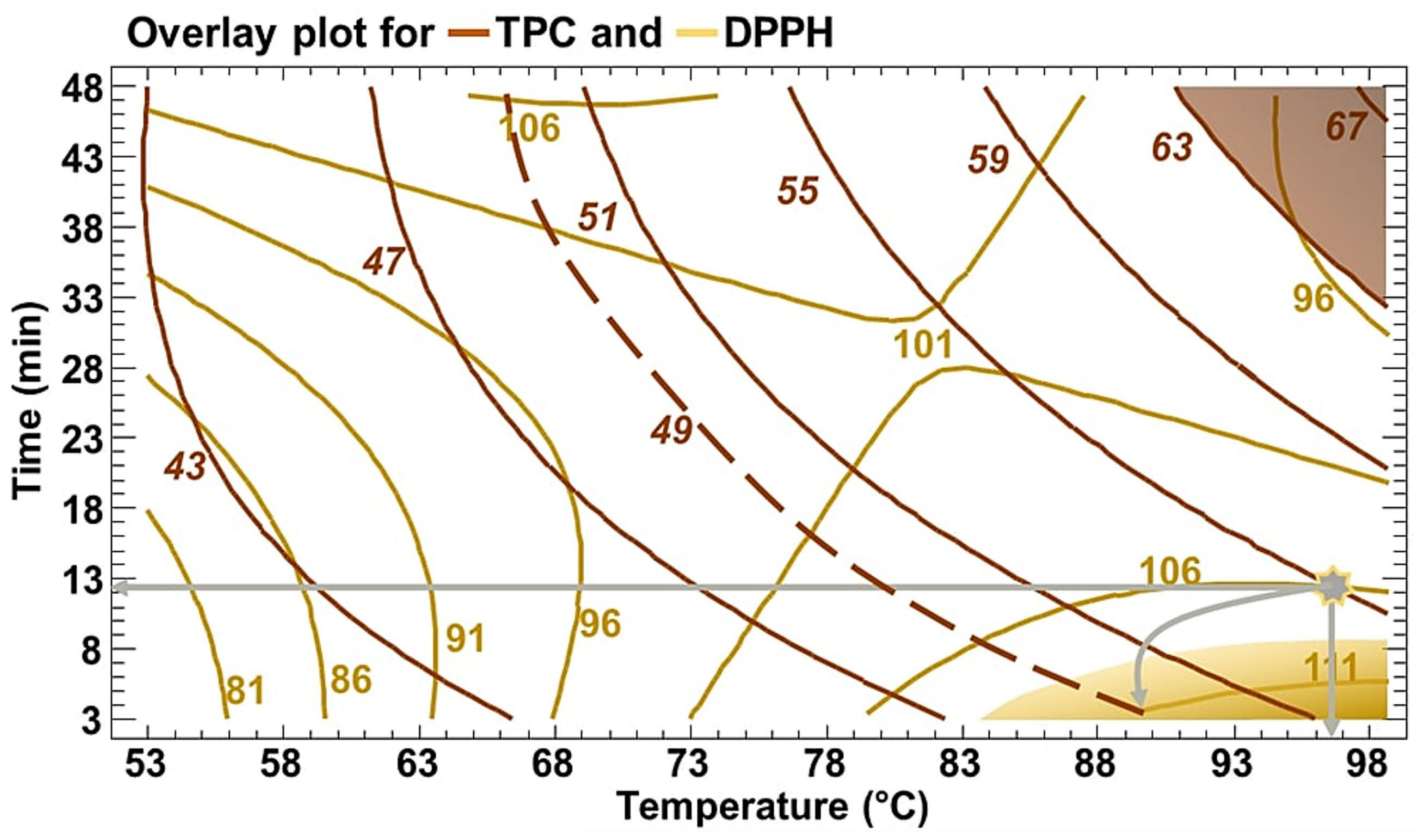
Overlay plot of TPC and DPPH activity of RTE as function of time and temperature.

To identify an optimal trade-off that satisfies both responses, an overlay plot (Figure 3) was generated. This analysis suggested combined conditions of 96.21 °C and 11.42 min, with predicted values of 56 mg GAE/g DM for TPC and 106.5 mg TE/g DM for DPPH.

Experimental validation was conducted to assess model robustness and to explore the feasibility of a shorter, more energy-efficient extraction condition. The validation results are presented in Table 4.

**Table 4:** Experimental validation of RSM-predicted optimum and alternative high-response conditions for TPC and DPPH activity in RTE.

Extraction parameters		TPC (mg GAE/g DM)		DPPH (mg TE/g DM)
Time (min)	Value obtained by assay	Value predicted by RSM	Value obtained by assay	Value predicted by RSM
11.42	59.73	56.16	102.66	106.55
10	49.94	54.30	101.90	103.50

At the overlay optimum (96.24 °C, 11.42 min), the experimentally measured TPC (59.73 mg GAE/g DM) and DPPH activity (102.66 mg TE/g DM) closely matched the predicted values, confirming the reliability of the RSM model. Additional validation at 90 °C for 10 min, which is also within the high-response region, produced TPC and DPPH values (49.94 mg GAE/g DM and 101.90 mg TE/g DM, respectively) that were both close to model predictions and comparable to those obtained at the overlay optimum. Given its proximity to the predicted optimum and its reduced time and energy requirements, this condition was selected as the optimal extraction point for subsequent analyses.

The TPC and DPPH obtained for the RTE in the present study fall within the range of values previously reported for red rooibos tea, although direct quantitative comparisons remain limited due to substantial methodological differences among studies. Variations in tea cultivar., fermentation degree, extraction temperature and time, solid-to-liquid ratio, and analytical expression units have been shown to strongly influence reported phenolic yields and antioxidant activity (13, 78–80).

Under traditional beverage preparation conditions, considerably lower phenolic contents and antioxidant activities have been reported. For instance, hot brewing of red rooibos tea at 95 °C for 20 min (1:17.5 *w/v*) resulted in a TPC of 5.36 mg GAE/mL and 73.05% DPPH inhibition, while cold brewing at 4 °C for 24 h yielded only 1.59 mg GAE/mL and 80.15% DPPH inhibition (81). Similarly, boiling extraction at 100 °C using a 1:25 *w/v* ratio for 1–10 min produced TPC values of 25–40 mg GAE/g, lower than those reported in this study, with limited improvement in antioxidant activity over time, suggesting rapid phenolic release followed by early equilibrium (82).

In contrast, the present study employed RSM-based optimization to identify extraction conditions (90 °C, 10 min, 1:60 *w/v*) that balance phenolic solubilization and thermal stability, without unnecessary resource use. Comparable optimum conditions (85 °C for 10 min) have been reported in studies evaluating time–temperature effects using univariate approaches (71).

### 3.3 UHPLC–QTOF–MS analysis for metabolic profile and polyphenolic composition of RTE

The phytochemical composition of RTE was profiled by UHPLC–QTOF–MS in both positive and negative ESI modes. In total, 43 metabolites were identified, including phenolic acids, flavonoids, fatty acids, coumarins, amino acids, and nitrogenous compounds. Positive and negative ionization modes detected 24 and 26 compounds, respectively. Seven metabolites, 1,2-benzenediol, gallic acid, vanillic acid, aesculetin, oleic acid, rutin, and isoorientin, were detected in both modes, indicating dual ionization behavior. For interpretation, the overlapping polyphenols were primarily reported from the negative ion mode, which is widely recognized as more sensitive for acidic phenolics and flavonoids (83, 84). An exception was made for isoorientin which exhibited higher signal intensity in positive mode, consistent with the known ionization behavior of C-glycosides (85).

Compounds contributing ≥2% of the total ion signal were classified as notable constituents, following previous practices (58), all shown in Table 5. Chromatograms of the compounds detected in RTE in the negative and positive ionization modes are presented in the Supplementary Figures S1, S2, respectively.

**Table 5:** Notable compounds detected by UHPLC–QTOF–MS in RTE (≥ 2%).

Compound name	Retention time	m/z ratio	Measured molar mass	ESI mode considered	Relative percentage (%) in respective ESI mode
Oleic acid	29.89	279.23133	280.23861	−	27.33
Isoorientin	7.15	449.1072	448.09992	+	23.57
Adenosine	1.31	268.10397	267.0968	+	19.77
Rutin	9.13	609.14568	610.15296	−	19.5
Myristic acid	27.82	227.20018	228.20746	−	13.07
Aesculetin	3.42	179.03393	178.0267	−	6.48
Aspalathin	7.1	453.139138	452.131313	+	6.02
9-Trans-Palmitelaidic acid	28.46	253.21579	254.22307	−	4.84
L-Glutamic acid	0.72	148.0607	147.05343	+	4.28
L-Tyrosine	0.77	182.08051	181.07323	+	4.22
1,2-Benzenediol (Catechol)	0.56	109.02933	110.0366	−	3.76
Caffeic acid	1.02	179.03399	180.04126	−	3.23
9Z,12Z-Linoleic acid	29.13	279.23133	280.23861	−	3.07
4-Hydroxycoumarin	1.48	163.04221	162.03493	+	2.84
Apigenin	15.46	269.04364	270.05092	−	2.72
Scopoletin	5.87	193.0495	192.04223	+	2.5
L-Leucine	0.81	132.10213	131.09486	+	2.4
Kaempferol	13.82	287.05487	286.04759	+	2.08

The flavonoid profile of the RTE was consistent with previous reports on red rooibos. Isoorientin (23.57%) is the predominant flavonoid, given its role as a major oxidation product of aspalathin during rooibos fermentation (8). Isoorientin is well recognized as a principal antioxidant flavone in rooibos, contributing to radical scavenging and cellular protection through hydrogen-donating and metal-chelating mechanisms (86). Rutin (19.5%) was also identified as a major constituent, in agreement with earlier studies describing it as one of the most abundant flavonol glycosides in red rooibos (87, 88), with a variety of bioactive properties (89).

Aspalathin (6.02%), a C-glycosylated dihydrochalcone unique to *A. linearis*, was also detected among the major phenolics. Although it is typically more abundant in green rooibos, its presence in the fermented extract may reflect incomplete oxidation during processing, allowing partial persistence alongside its flavone derivative, isoorientin (8). This finding suggests a balance between oxidative degradation and stabilization processes influenced by fermentation and extraction conditions, and is relevant given the reported antioxidant activities of aspalathin (90).

Kaempferol (2.08%) and apigenin (2.72%) were present in moderate amounts, consistent with their classification as secondary flavonoids in rooibos infusions (87). Kaempferol has been associated with modulation of key pathways involved in cancer development (91), while apigenin is recognized for its antioxidant, anti-inflammatory, and anticancer properties (92).

Among phenolic acids, caffeic acid (3.23%) was the predominant hydroxycinnamic acid detected. This compound is commonly formed through the degradation or transformation of higher-molecular-weight polyphenols during processing (8, 93) and is known for its radical-scavenging and anti-inflammatory activities (94). Catechol (1,2-benzenediol; 3.76%) was also identified at moderate levels; although not a typical native rooibos constituent, it likely originates from oxidative or thermal degradation of complex phenolics during fermentation or extraction (93, 95).

Coumarin derivatives, particularly aesculetin (6.48%), scopoletin (2.5%), and 4-hydroxycoumarin (2.84%), were detected in notable amounts. These compounds may arise from oxidative coupling of cinnamic acid derivatives and reflect downstream oxidative processes associated with fermentation and heat exposure (93, 96). Such coumarins have been reported to exhibit antioxidant, antibiofilm, and antimicrobial activities against both Gram-positive and Gram-negative bacteria (97).

In addition to phenolics, several fatty acids (oleic, myristic, palmitelaidic, and linoleic acids) were detected, consistent with previous reports showing that herbal teas may contain lipid-derived constituents originating mainly from plant cellular membranes and primary lipid metabolism, which can be released during processing and extraction (98, 99). Amino acids and nitrogenous compounds, including adenosine, L-glutamic acid, L-tyrosine, and L-leucine, were also present. Adenosine has been previously reported in metabolomic studies of teas and herbal infusions and is thought to arise from nucleotide degradation during fermentation and thermal processing (100). Free amino acids are likewise common in fermented plant products, where they contribute to flavor development and, in some cases, antioxidant properties (101).

### 3.4 Antibacterial efficacy of RTE

Inhibitory activity was observed against *S. aureus* and *P. aeruginosa* using the disc diffusion assay, with mean inhibition zones of 12 mm and 10 mm, respectively. No zones of inhibition were detected for *B. cereus*, *L. monocytogenes*, *E. coli*, or *S. typhimurium* under the tested conditions.

While data for comparison in the literature are limited, Almajano et al. (2008) reported modest inhibition of *B. cereus* (7 mm) using a brief hot-water infusion (boiling water for 5 min), whereas no activity was detected in their assays against *E. coli* or *P. aeruginosa* (31). Similarly, Salkić and Ćavar Zeljković (2015) observed no measurable antibacterial effects of rooibos infusions using paper disc diffusion method against *S. aureus*, *E. coli*, or *P. aeruginosa* (102). In contrast, the optimized aqueous extraction applied in the present study yielded a polyphenol-rich extract, which likely contributed to the measurable inhibition observed against *S. aureus* and *P. aeruginosa*, as opposed to other studies which used non-optimized parameters.

To further characterize antibacterial efficacy, MIC and MBC values were determined using the broth microdilution method, as shown in Table 6.

**Table 6:** MIC and MBC values of RTE against tested bacterial strains.

Bacterial strain	MIC (mg/mL)	MBC (mg/mL)
*S. aureus*	25	25
*L. monocytogenes*	25	50
*P. aeruginosa*	50	50
*E. coli*	50	100
*B. cereus*	ND*	ND
*S.* typhimurium	ND	ND

*ND, not detected.

RTE exhibited bacteriostatic and bactericidal activity against *S. aureus*, *L. monocytogenes*, *P. aeruginosa*, and *E. coli*, while *B. cereus* and *S. typhimurium* remained resistant at the tested conditions. The lowest MIC and MBC values were recorded for *S. aureus* (25 mg/mL). RTE showed an MIC of 25 mg/mL and an MBC of 50 mg/mL against *L. monocytogenes*. In contrast, Gram-negative strains were less susceptible, with MIC values of 50 mg/mL for both *P. aeruginosa* and *E. coli*, and MBC values of 50 mg/mL and 100 mg/mL, respectively.

Comparable quantitative MIC/MBC data for aqueous red rooibos extracts are scarce. However, Simpson et al. (2013) reported substantial growth inhibition of *S. aureus* (85%) and moderate suppression of *E. coli* (34%) using red rooibos infusions (boiling water for 5 min) in a broth-based assay, supporting the higher sensitivity of *S. aureus* to rooibos-derived compounds (24). The lower magnitude of inhibition in that study may reflect differences in phenolic concentration and extraction methodology.

The reduced susceptibility of Gram-negative bacteria observed in this study aligns with previous reports attributing this resistance to the presence of an outer membrane, reduced permeability, and active efflux systems (103, 104). Nevertheless, the detection of bactericidal effects at higher concentrations demonstrates that RTE retains appreciable activity even against these species.

The antibacterial effects of RTE are likely linked to its phenolic composition. Several identified metabolites have documented antimicrobial properties. Flavonoids such as rutin, apigenin, kaempferol, and isoorientin have been reported to disrupt membrane integrity, interfere with enzymatic activity, and inhibit bacterial virulence factors (105–108). Phenolic acids, particularly caffeic acid, are well known for their antibacterial activity through oxidative stress induction and membrane damage (108). Furthermore, coumarin derivatives, including aesculetin and scopoletin, have also been shown to exert broad-spectrum antimicrobial effects, including activity against *P. aeruginosa* (109). Catechol may further contribute via redox cycling and reactive oxygen species generation, leading to intracellular damage (108).

A notable difference was observed between the disc diffusion and broth microdilution results. While *E. coli* and *L. monocytogenes* showed measurable MIC and MBC values, no inhibition zones were detected on agar. This discrepancy is well documented and can be attributed to limited diffusion of large, polar, or glycosylated phenolics (e.g., rutin and isoorientin) through the agar matrix, resulting in underestimation of activity in diffusion-based assays (109). In contrast, broth assays enable direct contact between bacterial cells and the extract, providing a more sensitive assessment of antibacterial potential (109).

The absence of activity against *B. cereus* and *S. typhimurium* suggests that the concentration or bioavailability of active compounds was insufficient to overcome their defense mechanisms, which include cell wall impermeability, enzymatic degradation, and multidrug efflux systems (109, 110).

### 3.5 Anticancer potential of RTE

**Figure 4:**
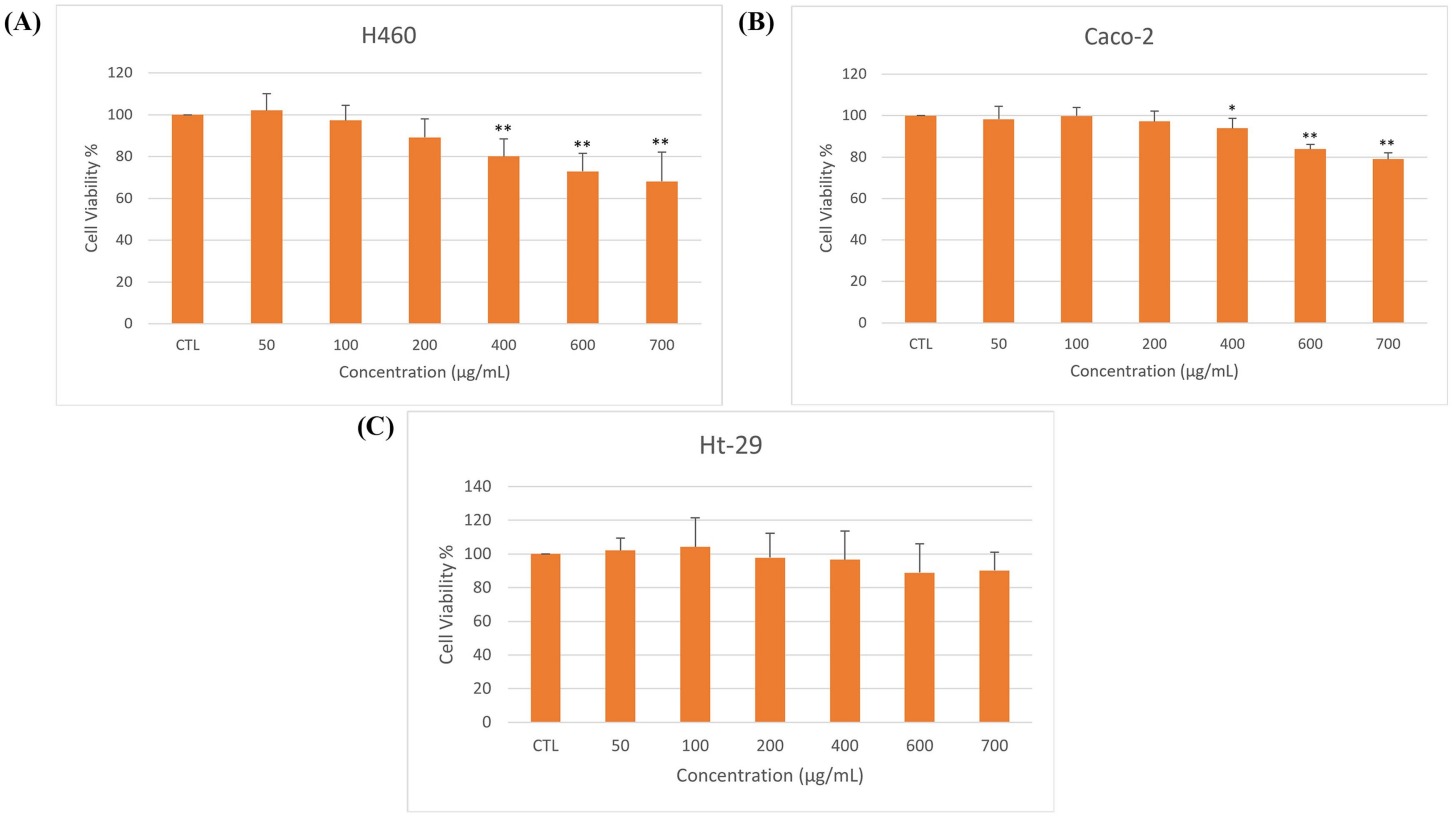
Effect of RTE on the viability of **(A)** H460, **(B)** Caco-2, and **(C)** HT-29 cells after 72 h of treatment. Data are expressed as mean ± SD (*n* = 3). *p* < 0.05 (*), *p* < 0.01 (**), compared with control group. Bars without asterisks are not significantly different from the control (*p* > 0.05).

The antiproliferative activity of RTE was assessed in H460, Caco-2, and HT-29 human cancer cell lines using the MTT assay following 72 h exposure to concentrations ranging from 50 to 700 μg/mL. A statistically significant reduction in cell viability was observed in Caco-2 and H460 cells, as seen in Figure 4.

In H460 cells (Figure 4A), RTE reduced cell viability by 31.86% at 700 μg/mL, with statistically significant effects observed starting at concentrations ≥ 400 μg/mL (*p* < 0.01). Similarly, Caco-2 cells (Figure 4B) exhibited a reduction in viability by 21.01% at 700 μg/mL, with statistically significant inhibition starting at ≥ 400 μg/mL (*p* < 0.05–0.01). In contrast, HT-29 cells (Figure 4C) showed no significant loss of viability even at the highest concentration tested (*p* > 0.05).

Notably, none of the cell lines reached 50% growth inhibition within the investigated concentration range, indicating limited cytotoxic potency under these tested conditions.

To the best of our knowledge, this study is the first to evaluate the effects of aqueous fermented rooibos tea extract on H460, HT-29, and Caco-2 cell lines using the MTT assay, and as a result, comparable reports of dose-dependent, modest cytotoxic effects of aqueous red rooibos extracts are scarce. However, Malibary (2024) observed significant inhibition in PC-3, HCT-116, and HepG_2_ cells at relatively high concentrations, with HepG_2_ cells showing the highest sensitivity to the rooibos extract with an IC_50_ of 1,399.41 ± 62.73 μg/mL, but cisplatin exhibited substantially greater potency (IC_50_ = 3.67 ± 0.25 μg/mL for HepG_2_ cells) (33). This suggests that although aqueous red rooibos extracts can suppress cancer cell viability, the high concentrations required limit their clinical relevance as direct cytotoxic agents.

These results may be related to the extract composition and assay characteristics. Aqueous extraction favors hydrophilic, largely glycosylated flavonoids such as isoorientin and rutin, which are recognized antioxidants but are not necessarily strong cytotoxins and may act through indirect mechanisms rather than acute cell killing (24, 111, 112). In addition, whole-extract testing may involve matrix effects that attenuate the activity of minor bioactive constituents, while the MTT assay itself can be influenced by metabolic and redox modulation by polyphenols. Differences in intrinsic cell-line sensitivity may further explain the greater responsiveness of H460 and Caco-2 cells compared with the more resistant HT-29 model (113).

## 4 Conclusion

In this study, an aqueous extraction protocol for red rooibos tea was systematically optimized using RSM, yielding an experimentally validated optimum at 90 °C for 10 min. Under these conditions, the extract exhibited a TPC of 49.94 mg GAE/g DM and DPPH of 101.90 mg TE/g DM. Comprehensive UHPLC–QTOF–MS profiling in both positive and negative ionization modes enabled the identification of 43 metabolites, with isoorientin (23.57%) and rutin (19.5%) emerging as prominent constituents of the RTE. Biological evaluation demonstrated selective antibacterial activity, with measurable inhibition zones against *S. aureus* and *P. aeruginosa* in disc diffusion assays and confirmed bacteriostatic and bactericidal effects against *S. aureus*, *L. monocytogenes*, *P. aeruginosa*, and *E. coli* in broth microdilution assay. In contrast, *B. cereus* and *S. typhimurium* remained resistant under the tested conditions. Anticancer screening using the MTT assay revealed statistically significant dose-dependent reductions in cell viability in H460 and Caco-2 cell lines at higher concentrations, while HT-29 cells were largely unresponsive, and IC_50_ values were not achieved across all three tested cell lines. One limitation of this study is that a single commercial red rooibos product was tested, which prompts the need for future studies to examine raw material variability. Additionally, future work may address *in vivo* relevance and underlying mechanisms of action, while also comparing red and green rooibos and exploring alternative extraction strategies to further define its functional and health-related applications. Overall, these findings position red rooibos tea as a chemically rich matrix whose bioactivity can be meaningfully enhanced through extraction optimization, supporting its potential use as a standardized antioxidant source with biological effects.

## Data Availability

The original contributions presented in the study are included in the article/Supplementary material, further inquiries can be directed to the corresponding author.
