# Supplementary material for: Optimized extraction of polyphenols from rooibos tea (Aspalathus linearis) and their biological activities
Source: Front Nutr. 2026 Mar 6;13:1778749. doi: 10.3389/fnut.2026.1778749 (PMC13002457; doi:10.3389/fnut.2026.1778749)
Supplement: Supplementary file 1 [file Table_1.docx]

**Supplementary Material**

| **Table S1.** Standards used for UHPLC–QTOF–MS analysis of RTE. |
| --- |
| \| **No** \| **Standard Name** \| \| --- \| --- \| \| 1 \| Oxalic acid \| \| 2 \| 1,2-Benzenediol \| \| 3 \| 1,2,3-Benzenetriol \| \| 4 \| 2-Methoxy-4-vinylphenol \| \| 5 \| 2,5-Dihydroxybenzoic acid \| \| 6 \| p-Coumaric acid \| \| 7 \| Vanillic acid \| \| 8 \| Gallic acid \| \| 9 \| Aesculetin \| \| 10 \| Caffeic acid \| \| 11 \| Starch \| \| 12 \| Ferulic acid \| \| 13 \| Myristic acid \| \| 14 \| Formononetin \| \| 15 \| 9-Trans-Palmitelaidic acid \| \| 16 \| Apigenin \| \| 17 \| 9Z,12Z-Linoleic acid (NMR) \| \| 18 \| Oleic acid \| \| 19 \| Catechin \| \| 20 \| 7,4'-Dimethoxy-3-hydroxyflavone \| \| 21 \| (Z)-3-Hydroxyoctadec-7-enoic acid (NMR) \| \| 22 \| Hispidulin \| \| 23 \| Quercetin \| \| 24 \| Isoorientin \| \| 25 \| Hyperoside \| \| 26 \| Rutin \| \| 27 \| L-Proline \| \| 28 \| Benzoic acid \| \| 29 \| Nicotinic acid \| \| 30 \| L-Leucine \| \| 31 \| Protocatechuic aldehyde \| \| 32 \| L-Glutamic acid \| \| 33 \| 4-hydroxycoumarin \| \| 34 \| L-Tyrosine \| \| 35 \| Scopoletin \| \| 36 \| Gallic Acid Ethyl Ester \| \| 37 \| Adenosine \| \| 38 \| Kaempferol \| \| 39 \| 7,3'-Dimethoxy-5,6,4'-trihydroxyisoflavone \| \| 40 \| Carnosic acid \| \| 41 \| Gibberellic acid \| \| 42 \| Aspalathin \| \| 43 \| Nothofagin \| |

| **(A)** |  | **(B)** |  |
| --- | --- | --- | --- |
| **(C)** | 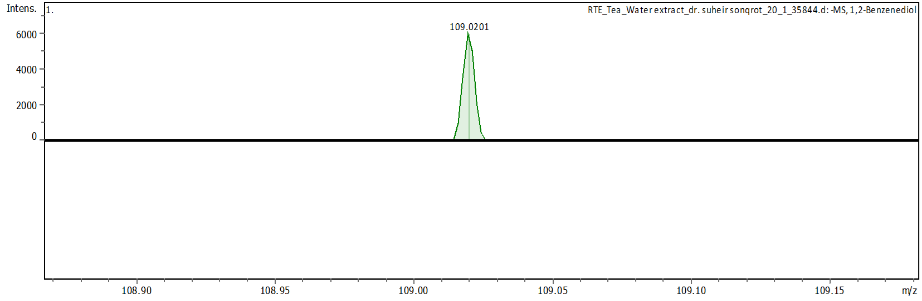 | **(D)** |  |
| **(E)** |  | **(F)** |  |
| **(G)** |  | **(H)** |  |
| **(I)** |  | **(J)** |  |
| **(K)** |  | **(L)** | 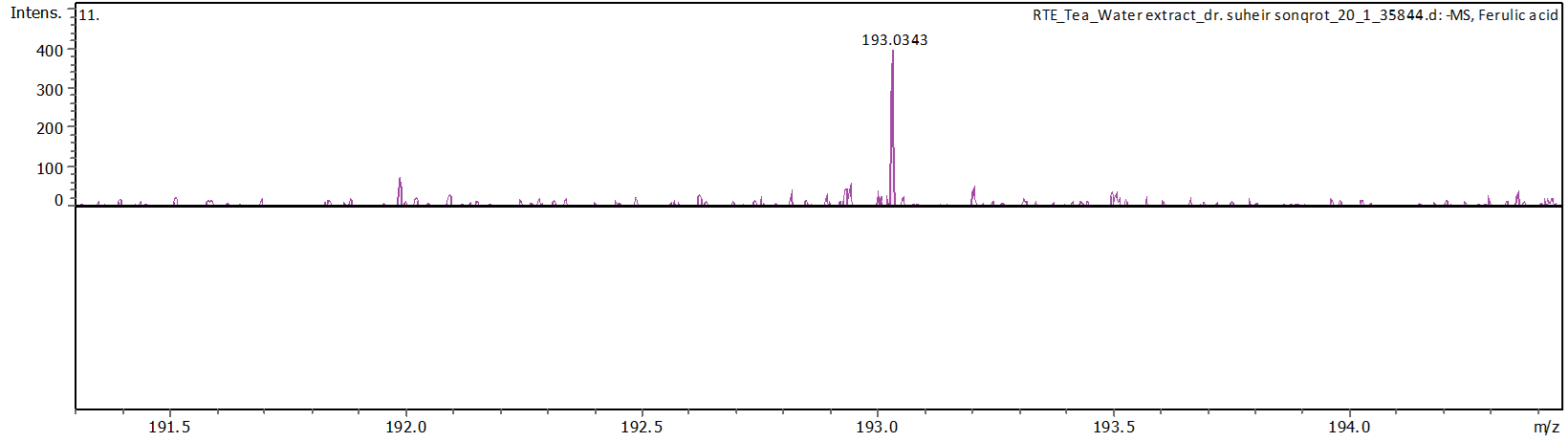 |
| **(M)** |  | **(N)** |  |
| **(O)** |  | **(P)** |  |
| **(Q)** |  | **(R)** |  |
| **(S)** |  | **(T)** |  |
| **(U)** |  | **(V)** |  |
| **(W)** |  | **(X)** |  |
| **(Y)** |  | **(Z)** |  |
| **Figure S1.** UHPLC–QTOF–MS chromatograms for the compounds detected in RTE in the negative ionization mode: **(A)** Oxalic acid, **(B)** 2,5-Dihydroxybenzoic acid, **(C)** 1,2-Benzenediol, **(D)** p-Coumaric acid, **(E)** 1,2,3-Benzenetriol, **(F)** Vanillic acid, **(G)** 2-Methoxy-4-vinylphenol, **(H)** Gallic acid, **(I)** Aesculetin, **(J)** Caffeic acid, **(K)** Starch, **(L)** Ferulic acid, **(M)** Myristic acid, **(N)** Formononetin, **(O)** Palmitelaidic acid, **(P)** Apigenin, **(Q)** 9Z,12Z-Linoleic acid, **(R)** Oleic acid, **(S)** Hispidulin, **(T)** Catechin, **(U)** Quercetin, **(V)** Isoorientin, **(W)** Hyperoside, **(X)** Rutin, **(Y)** 7,4'-Dimethoxy-3-hydroxyflavone, and **(Z)** (Z)-3-Hydroxyoctadec-7-enoic acid. | | | |

| **(A)** |  | | **(B)** |  |
| --- | --- | --- | --- | --- |
| **(C)** |  | | **(D)** |  |
| **(E)** |  | | **(F)** |  |
| **(G)** |  | | **(H)** |  |
| **(I)** |  | | **(J)** |  |
| **(K)** |  | | **(L)** |  |
| **(M)** |  | | **(N)** |  |
| **(O)** |  | | **(P)** |  |
| **(Q)** |  | | **(R)** |  |
| **(S)** |  | | **(T)** |  |
| **(U)** |  | | **(V)** |  |
| **(W)** | |  | | |
| **Figure S2.** UHPLC–QTOF–MS chromatograms for the compounds detected in RTE in the positive ionization mode: **(A)** 1,2-Benzenediol, **(B)** L-Proline, **(C)** Benzoic acid, **(D)** Nicotinic acid, **(E)** Protocatechuic aldehyde, **(F)** L-Glutamic acid, **(G)** 4-hydroxycoumarin, **(H)** Vanillic acid, **(I)** Gallic acid, **(J)** Aesculetin, **(K)** L-Tyrosine, **(L)** Scopoletin, **(M)** Gallic acid ethyl ester, **(N)** Adenosine, **(O)** Oleic acid, **(P)** Kaempferol, **(Q)** 7,3'-Dimethoxy-5,6,4'-trihydroxyisoflavone, **(R)** Carnosic acid, **(S)** Gibberellic acid, **(T)** Isoorientin, **(U)** Rutin, **(V)** Nothofagin, and **(W)** Aspalathin. | | | | |
